# Penicillin Allergy Evaluation and Delabeling in Nursing Homes: An Implementation Science-Guided, Multi-stakeholder Qualitative Study

**DOI:** 10.1093/ofid/ofag693

**Published:** 2026-10-23

**Authors:** Kap Sum Foong, Amy M LeClair, Majd Alsoubani, Shira Doron, Alysse G Wurcel

**Affiliations:** Department of Medicine, Division of Infectious Diseases, Denver Health, Denver, Colorado, USA; Division of Geographic Medicine and Infectious Diseases, Tufts Medical Center, Boston, Massachusetts, USA; Department of Medicine, Tufts Medical Center, Boston, Massachusetts, USA; Division of Geographic Medicine and Infectious Diseases, Tufts Medical Center, Boston, Massachusetts, USA; Division of Geographic Medicine and Infectious Diseases, Tufts Medical Center, Boston, Massachusetts, USA; Department of Medicine, AdventHealth, Orlando, Florida, USA; Section of General Internal Medicine, Boston Medical Center, Boston, Massachusetts, USA

**Keywords:** antimicrobial stewardship, delabel, nursing home, penicillin allergy, qualitative

## Abstract

**Background:**

Penicillin allergy labels (PALs) are common and often inaccurate, contributing to suboptimal antibiotic prescribing and adverse outcomes. These challenges are particularly pronounced in nursing homes (NH), where PAL prevalence and antibiotic exposure are high, yet penicillin allergy evaluation and delabeling are rarely implemented and stakeholder perspectives are poorly understood. We sought to identify barriers and facilitators to implementing penicillin allergy evaluation and delabeling in NH, incorporating resident, caregiver, and healthcare professional (HCP) perspectives.

**Methods:**

Between July and December 2025, we conducted semi-structured interviews with NH residents, a caregiver, and HCPs across 5 Massachusetts facilities. Interview guides were informed by the updated Consolidated Framework for Implementation Research. Data were analyzed using rapid thematic analysis with a structured matrix approach.

**Results:**

Twenty-five participants (13 residents, 1 caregiver, 11 HCPs) were included. Five cross-cutting themes emerged: (1) PALs persist unquestioned, (2) skepticism about labels coexists with reluctance to delabel, (3) structural barriers are decisive, (4) feasibility is facility- and method-specific, and (5) education and trust enable engagement, but receptiveness cannot be assumed. HCPs cited residents’ cognitive impairment as a barrier, yet 13 of 21 residents screened could provide meaningful histories and participate in decision-making. History-based assessment was considered most feasible, whereas views on direct oral challenge and skin testing were divided.

**Conclusions:**

Many NH residents can engage in penicillin allergy evaluation; capacity should be assessed individually. Implementation is constrained primarily by structural barriers rather than lack of interest. Low-resource, history-based, trust-building strategies may support adoption in NH.

Penicillin allergy is the most commonly reported drug allergy, affecting approximately 10% of the US population [1]. The vast majority of individuals with penicillin allergy labels (PALs) are not truly allergic, with up to 95% able to tolerate beta-lactam antibiotics [1]. People with PALs are more likely to receive broad-spectrum and second-line antibiotics that are less effective and associated with increased risks of adverse drug reactions, *Clostridioides difficile* infection (CDI), and antimicrobial resistance [2, 3]. Penicillin allergy evaluation and delabeling are evidence-based interventions for these challenges [4]. Structured approaches, including detailed allergy history assessment, direct oral challenge, and penicillin skin testing, are safe and effective in identifying patients who can tolerate beta-lactam antibiotics [3, 4].

Delabeling initiatives have been successfully implemented across inpatient and ambulatory settings and are increasingly integrated into antimicrobial stewardship programs, improving antibiotic selection and reducing reliance on broad-spectrum agents [5–8]. Implementation of delabeling programs in nursing homes (NH), however, is not routine [5, 9–11]. Approximately 50%–75% of NH residents receive at least one course of antibiotics annually, and nearly 1 in 4 NH residents carries a documented PAL, substantially higher than in the general population [12, 13]. This combination of high antibiotic use and unverified PALs leads to increased use of antibiotics associated with higher CDI risk and increased risk of adverse drug events from second- or third-line antibiotic agents [14, 15].

Prior qualitative work has explored barriers and facilitators to penicillin allergy verification in the NH setting. However, this study was limited to the Veterans Affairs (VA) population [16]. There remains limited understanding of stakeholder perspectives on penicillin allergy evaluation and delabeling in diverse NH settings outside of the VA network. To diversify representation of NH stakeholder voices, we used implementation science-guided methods to contextualize perceptions, barriers, and facilitators to implementation of penicillin allergy evaluation and delabeling in Massachusetts NH settings.

## METHODS

### Study Setting and Participants

We conducted a qualitative study using semi-structured interviews with residents, caregivers, and healthcare professionals and administrators (collectively HCPs), to identify barriers and facilitators to implementation of penicillin allergy delabeling in NH setting. This study is reported in accordance with the Consolidated Criteria for Reporting Qualitative Research (COREQ) (Supplementary Appendix 1) [17].

We built on an established academic-public health partnership between our research team, the Massachusetts Department of Public Health, and Massachusetts NH. This partnership developed through prior collaborative work on antibiotic allergy prevalence and the statewide Antibiotic Start Reporting Program, a multifaceted antimicrobial stewardship initiative in NH settings [13, 18]. This existing partnership facilitated NH engagement, participant recruitment, and study implementation.

The Massachusetts Department of Public Health distributed study information through their NH listservs, and the study team (K. S. F.) subsequently conducted direct outreach to interested NH. Facility leadership provided permission for recruitment within participating NH. We then recruited participants and screened interested individuals for eligibility before scheduling interviews. We used purposive sampling to recruit 3 stakeholder groups: (1) NH residents with a documented PAL, (2) caregivers of such residents, and (3) NH HCPs, including physicians, advanced practice providers, nurses, nursing directors, pharmacists, and administrators. Eligible participants were ≥18 years old. Residents either provided informed consent themselves or participated through a caregiver if cognitive impairment limited their ability to consent. Healthcare professionals needed experience caring for NH residents with penicillin allergies. This group intentionally included facility leadership, nursing directors, and administrators, alongside frontline clinical staff, because they represent important stakeholders shaping implementation.

### Interview Guide Development

We based the interview guides on the updated Consolidated Framework for Implementation Research (CFIR), drawing from prior related work [19], and focused on domains related to individuals, inner setting, outer setting, innovation, and implementation process [20]. We selected these domains because they capture key determinants of delabeling, including stakeholder knowledge and beliefs, organizational resources and workflows, external influences, and implementation processes within NH settings. We developed 3 semi-structured interview guides tailored to each stakeholder group (Supplementary Appendix 2). We began each interview by exploring participants’ understanding of and experiences with PAL, including perceptions of allergy validity and impact on care. We then provided standardized information about PAL and penicillin allergy evaluation and asked participants about perceived benefits, risks, reactions, and willingness to pursue testing. We subsequently explored barriers, facilitators, and implementation strategies, with additional questions for HCPs regarding organizational context and implementation readiness. We concluded each interview by asking participants whether there were additional perspectives or experiences they wished to share. The guides were piloted with internal team members, after which we refined wording, reordered items, and added probes. We intentionally elicited these baseline perceptions before providing any standardized information, to reduce priming of subsequent responses.

### Data Collection

Between July and December 2025, KSF conducted in-person semi-structured interviews in private spaces within participating NH. Interviews lasted approximately 30–45 minutes. Participants provided verbal informed consent prior to participation. Interviews were audio-recorded with permission and professionally transcribed. The lead interviewer-analyst (K. S. F.) is an infectious disease physician with a professional interest in antimicrobial stewardship and penicillin allergy delabeling, which could shape interpretation. To mitigate this, interviews used open-ended prompts, and baseline perceptions were elicited before information was provided. When interviewing residents, the interviewer (K. S. F.) introduced himself as a researcher rather than a clinician and emphasized that participation was voluntary and would not affect care.

### Data Analysis

Guided by updated CFIR framework [20], we developed an analytic matrix template for data reduction and analysis (Supplementary Appendix 3). We used a rapid thematic analysis approach to systematically code interview data and identify responses most relevant to implementation strategy development. This approach generates timely, actionable insights for intervention design while maintaining methodological rigor comparable to traditional qualitative methods [21]. Our analysis was primarily deductive using an a prior analytic matrix of codes and subcodes derived from CFIR domains and constructs, as well as the interview guide. The initial matrix was developed by the lead analyst (K. S. F.) and refined with the analytic team (A. M. L., M. A., A. G. W.) before application. Data that did not fit the prespecified codes and subcodes were retained in an open category, reviewed inductively by the team, and, where recurrent, incorporated as new codes and subcodes by consensus.

To support interpretation, we operationalized the CFIR domains for penicillin allergy evaluation and delabeling in NH as follows. Innovation refers to the delabeling approaches themselves (history-based risk stratification, direct oral challenge, and penicillin skin testing) and their perceived evidence base, relative advantage, complexity, and adaptability. Individuals refers to the roles involved, including NH residents and caregivers (innovation recipients), HCPs who would deliver or lead delabeling (innovation deliverers and implementation leads), and facility and corporate leaders (high-level leaders), and to their need, capability, and motivation. Inner setting refers to the NH itself, including staffing and workflows (structural characteristics), competing priorities (relative priority), fit with existing processes (compatibility), access to training (access to knowledge and information), and available resources. Outer setting refers to external entities with which the NH is networked, including discharging hospitals and third-party pharmacy and provider services (partnerships and connections). Implementation process refers to the activities required to put delabeling into practice, including planning, assigning roles (teaming), and engaging residents, families, and staff. Because themes were cross-cutting, a single theme could map to more than one domain.

Shortly after each interview, the lead interviewer-analyst (K. S. F.) summarized participant responses, extracted representative quotations, and coded all transcripts. A second analyst (M. A.) independently reviewed coding against source transcripts for all interviews to ensure accuracy. Analytic decisions were discussed with a multidisciplinary team including a non-physician medical sociologist (A. M. L.). We did not calculate a formal inter-coder agreement statistic. The analytic team then iteratively refined themes through discussion and resolved discrepancies by consensus, with reference to source transcripts as needed.

## RESULTS

Of 103 NH in the metropolitan Boston area that were invited to participate, 5 permitted recruitment. Participating facilities were a mixture of independent and chain-affiliated, ranging from 80 to 190 beds. Most (4/5) used an off-site, third-party pharmacy model and lacked an on-site allergist, infectious disease physician, or dedicated antimicrobial stewardship pharmacist. Among 36 individuals screened, 25 completed interviews, including 13 residents, 11 HCPs, and 1 caregiver. Of the 21 residents screened, 13 (62%) were able to describe their penicillin allergy history and engage meaningfully in discussions regarding evaluation. The remaining 8 residents were unable to engage in meaningful communication. Among HCPs, 3 declined participation because they were not interested. Resident and caregiver participants were predominantly older adults, with the majority aged >60 years, and were primarily men (64%) and White (79%). Healthcare professionals were primarily women (64%) and represented a range of clinical and administrative roles. Additional demographic characteristics are shown in Table 1.

**Table 1.** Characteristics of Study Participants by Stakeholder Group

Characteristic	Residents^a^, N = 14	HCPs^b^, N = 11
Age, n (%)		
≤60	3 (21)	10 (91)
61–70	4 (29)	1 (9)
71–80	5 (36)	0
81–90	2 (14)	0
Gender n (%)		
Female	5 (36)	7 (64)
Male	9 (64)	4 (36)
Race, n (%)		
White	11 (79)	4 (36)
Black	2 (14)	5 (45)
Asian	1 (7)	2 (18)
Ethnicity, n (%)		
Non-Hispanic	13 (93)	11 (100)
Hispanic	1 (7)	0

Abbreviation: HCP, health care professional.

^a^Residents included residents (13) and a caregiver.

^b^HCPs included a physician, advanced practice providers (2), nurses (4), a pharmacist, nursing directors (2), and an administrator.

Five cross-cutting themes emerged across stakeholder groups. Each theme, with its corresponding CFIR domains and constructs and representative quotations from residents, the caregiver, and HCPs, is presented in Table 2. Figure 1 summarizes the current state and the stakeholder-identified barriers and facilitators.

**Figure 1.**
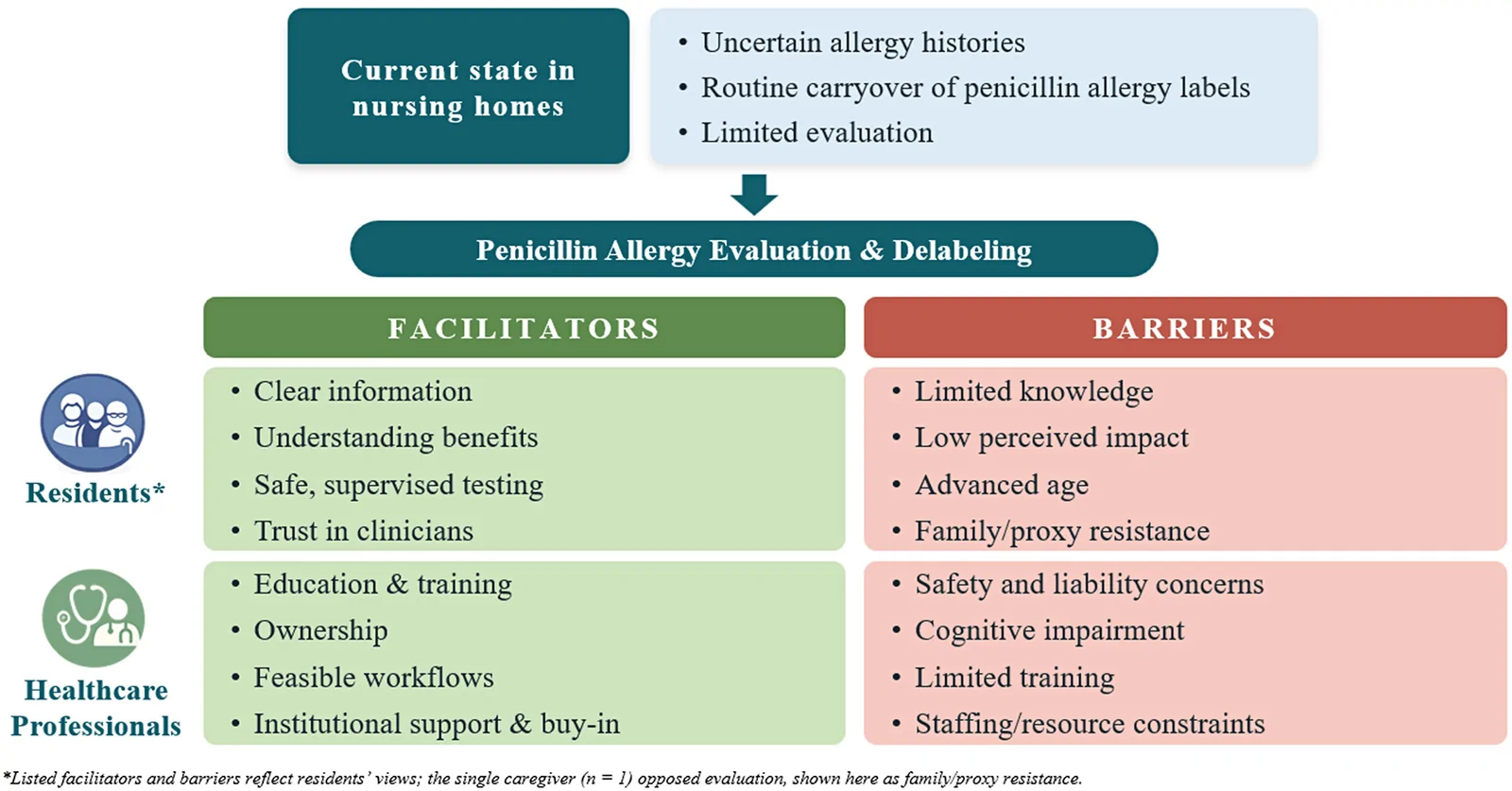
Stakeholder-identified barriers and facilitators to penicillin allergy evaluation and delabeling in nursing homes.

**Table 2.** Barriers and Facilitators to Penicillin Allergy Evaluation and Delabeling in Nursing Home, Mapped to Updated CFIR Domains and Constructs, by Stakeholder Group

Themes	CFIR Domain (Construct)	Resident^a^ Perspectives	HCP Perspectives
(1) Penicillin allergy labels persist unquestioned	Innovation (evidence base, relative advantage); individuals (innovation recipients: capability, need); outer setting (partnerships and connections)	*I was told as a child by my birth mother. She didn’t go into detail about it. I've never tried penicillin. I don’t know for a fact that I am actually allergic.* (Resident ID #6) *… they almost always ask about allergies, and I say penicillin, and that's it. It never really gets discussed again… I’ve obviously had infections along the line, and they found another way to treat me without using penicillin.* (Resident ID #7) *I’m indifferent to it… even if it's not a real one, I know that there are alternatives, so I don’t have to be concerned.* (Caregiver ID #25)	*Whatever comes to us from the hospital is the guidelines for medications and allergies. We pretty much follow that completely until something actually happens in house.* (Nurse ID #10) *…the people doing admissions and intake don’t have the knowledge or the investment in it to really look much harder than just a copy the words…* (Nursing director ID #23)
(2) Skepticism about labels coexists with reluctance to delabel	Individuals (innovation recipients and deliverers: motivation, capability); innovation (evidence base, relative advantage); inner setting (available resources)	*Well, I wonder if it really exists… I’d be interested, but I don’t know whether I want to put myself in jeopardy.* (Resident ID #6) *If it was the only antibiotic on the planet, then yeah, of course you’d have to. There's a million out there. I don’t have to expose myself to penicillin.* (Resident ID #18)	*We are over-documenting that people are allergic to certain medications, but in reality, they are not really allergic to many of those things.* (Nurse ID #13) *When a patient has a penicillin allergy label… I trust it, and I don't test it. I don't do anything. If they need an antibiotic, I will prescribe something that is safe.* (Physician ID #1) *It [penicillin allergy evaluation] doesn’t always happen… I don’t know if it's liability or being overcautious.* (Pharmacist ID #2) *It's nerve-racking in this setting, because I just don't have access to emergency care if somebody goes into anaphylactic shock…* *Is it going to happen? Probably not.* (APP ID #3) *I think it's up to the doctors, because I don’t want to take a chance. What if they’re allergic to it, right?* (Nurse ID #22) *…some are dementia patient, some have psychosis, and some of them have Alzheimer's, so they're not going to remember their allergy history*. (Nurse ID #14)
(3) Structural barriers are decisive	Inner setting (structural characteristics, relative priority, available resources); implementation process (planning, teaming); individuals (high-level leaders, implementation leads); outer setting (partnerships and connections)	Not applicable	*I can’t imagine just looking at all the stuff we do here. If I see one more thing I have to do, I’m gonna scream.* (Nurse ID #10) *There is no process. It needs a process… There should be a set process of whose job it is to ask, to reconcile those allergies, to make sure that what is documented is what's supposed to be. Clear roles and expectations.* (Physician ID #1) *It is good to have a role model spearheading some of the quality improvement projects. I think the infection control nurses could be the champion. They are available in every facility.*” (APP ID #26) *In nursing home, almost everything is third party… Our pharmacy is also third party. So if something happens and they don’t necessarily talk to each other, so that's a pretty big gap in terms of how things get dropped or missed… They come in once a month… They're covering multiple facilities.* (Nurse ID #22) *If you’re referring them from here to somewhere else, the facility needs to arrange somebody to go with them to monitor them. There are certain patients that you know, if they are cognitively impaired, they need somebody to go with them. So that may be challenging for us.* (Nursing director ID #23) *There is a lot of flag fatigue, so I think it would be kind of blown over more than it would be addressed, to be honest.* (Pharmacist ID #2) *…the liaison would have to talk to my corporate overlord. Does the corporate want it? Are they willing to undergo what they see as risk?* (Nursing director ID #4)
(4) Feasibility is facility- and method-specific	Innovation (complexity, adaptability); inner setting (structural characteristics, available resources, compatibility)	Not applicable	*Including a structured penicillin allergy history taking during the admission workflow can be a big step toward solving this problem. It would be a good idea if you can start doing that once they start admitting here, even if they come from the hospital … reassessing and asking those questions can be helpful.* (Physician ID #1) *…in terms of skin testing, you know, everybody knows that if your skin turns red and starts swelling, something's wrong. So it can be really easy for not only the nurses to monitor, but the patients as well. It is safer.* (Nursing director ID #4) *It [penicillin skin testing] would not be feasible here. There's just no staff available to support it.* (Pharmacist ID #2) *When we start a new antibiotic, we are already closely monitoring the patient for a reaction… if they have a rash or something minor, I give Benadryl. If it is more severe, we can give an EpiPen injection.* (APP ID #3)
(5) Education and trust enable engagement, but receptiveness cannot be assumed	Individuals (Individuals (innovation recipients: need, capability); inner setting (access to knowledge and information); implementation process (engaging)	*If I understood what the results were, if there was any danger, I might not go through the testing. But, if it wasn’t a very risky thing, I would do it.* (Resident ID #5) … *being in a supervised setting makes all the difference, and if something goes wrong, people can react.* (Resident ID #12) *I’d like to know about myself a little more. I’m open to [the testing], I could not be allergic and not even know it.* (Resident ID #17) *If my doctor recommends it, I would trust their experience and do it.* (Resident ID #8) *If I were 35 or 40, maybe even 50, I would say I’ll try it. But I am 77. I’m not going to live a whole lot longer, so why take a chance?* (Resident ID #7)	*Anything that we do in a setting like this, we just make sure that people are educated about it… Once they know what and why we’re doing it, they will most likely be more equipped to do it.* (Nursing director ID #23) *We can prescribe more penicillin, which is not too broad spectrum, so we can prevent resistance instead of choosing stronger guns. Maybe most people don’t really have a penicillin allergy.* (APP ID #26) *…you can walk up to somebody and tell them you need this. This is good for you. But if you don’t have that level of trust… then you’re dealing with a position of mistrust.* (Administrator ID #16)

Abbreviations: APP, advanced practice provider; CFIR, Consolidated Framework for Implementation Research; EHR, electronic health record; HCP, health care professional.

^a^Includes quotations from residents and a single caregiver.

### Theme 1: PALs Persist Unquestioned

Residents frequently reported vague, remote, or secondhand allergy histories originating in childhood and often lacked details about the original reaction. Many were uncertain whether they remained truly allergic, had rarely been reassessed, and were unaware that a label could be evaluated or removed. For most, PALs simply persisted unchallenged because an alternative was always found, and the caregiver similarly treated the label as settled and inconsequential given available alternatives. Healthcare professionals mirrored this passivity, describing allergy documentation as an admission-based, record-driven task led by nursing staff and anchored to hospital discharge paperwork, with little systematic verification, and it rarely extended to the resident.

### Theme 2: Skepticism About Labels Coexists With Reluctance to Delabel

Residents reported little prior awareness of penicillin allergy evaluation or delabeling. Some questioned whether their label was still accurate yet hesitated to pursue evaluation, citing fear of a reaction and the readily available alternative antibiotics, which made the label seemed low-stakes. Among HCPs, recognition that penicillin allergy is frequently overdiagnosed did not translate into delabeling. Concerns about patient safety, liability, and responsibility for adverse outcomes drove default avoidance of verification. Hesitancy persisted even after a tolerated dose and, for some, was heightened by the setting's limited access to emergency care. Healthcare professionals also cited cognitive impairment as a reason for hesitancy, describing residents with dementia or related conditions as unable to provide a reliable allergy history or to take part in decision-making.

### Theme 3: Structural Barriers Are Decisive

Beyond individual attitudes, HCPs described structural constraints as the decisive determinant of whether evaluation occurs. Staffing shortages, turnover, and competing clinical priorities limited the capacity to absorb new tasks. Most prominent was the absence of a defined process and clear ownership, with providers disagreeing about who should be responsible for reconciling and removing labels. Several proposed assigning ownership to a defined champion, often an existing role such as an infection control nurse, integrated into established workflow. Facility infrastructure compounded these barriers, including off-site, third-party pharmacy models, and the transportation and monitoring demands of external referral, particularly for cognitively impaired residents. Healthcare professionals were skeptical that electronic health record prompts alone would be sufficient, citing alert fatigue, documentation burden, and turnover, and several favored onsite or embedded approaches over external referral. Implementation was also seen as contingent on institutional and corporate buy-in.

### Theme 4: Feasibility Is Facility- and Method-Specific

Healthcare professionals consistently viewed history-based assessment as the most feasible entry point because it could be embedded in the admission workflow. Views on definitive testing diverged along facility resources and monitoring capacity. Some favored skin testing as safer and more controlled and easily monitored by both staff and residents. Others judged skin testing infeasible for lack of supporting staff and considered direct oral challenge more practical because residents started on antibiotics are already closely monitored.

### Theme 5: Education and Trust Enable Engagement, but Receptiveness Cannot Be Assumed

Despite limited baseline knowledge, residents were generally receptive to evaluation when given clear information regarding risks and benefits and when they felt safe and monitored during testing. Many would proceed if the evaluation were recommended by a trusted clinician. Willingness was also shaped by information needs and life context; several older residents weighed evaluation against limited life expectancy, whereas others expressed curiosity about clarifying their allergy status regardless of age. Healthcare professionals were similarly open to education, frequently acknowledging their own knowledge gaps and viewing training as a prerequisite to engagement. Several also connected delabeling to better prescribing and stewardship. Many believed residents and families would be receptive if educated and involved and that delabeling could be reassuring once its benefits were understood. At the same time, HCPs cautioned that receptiveness cannot be assumed as families or healthcare proxies may resist evaluation because of prior experiences or attachment to the label, making engagement and consent essential, particularly for residents with cognitive impairment.

### Divergent Perspectives

Not all participants shared these views. A minority of residents clearly recalled prior reactions, including anaphylaxis, and were confident their labels were accurate and did not warrant reassessment. Resident ID#20 stated, “*I went into anaphylactic shock… It was very serious… I’m still scared to death. I wouldn’t go near it… I just don’t see any reason. Why go looking for trouble*.” Among HCPs, a minority regarded documented PALs as generally accurate and reliable: “*Things can happen, but typically nine out of ten, it's always correct*” (Nursing director ID#4).

## DISCUSSION

This study examined how NH residents, their caregiver, and HCPs perceive PAL and its evaluation and delabeling, and it identified barriers and facilitators along the pathway from a persistent, uncertain allergy label toward potential delabeling. One finding with direct implications for feasibility concerns residents’ capacity to participate. Although many residents carried uncertain or poorly documented histories of the kind that warrant evaluation, more than half of those were nonetheless able to discuss their allergy and were conditionally willing to proceed. At the same time, HCPs repeatedly identified cognitive impairment as a barrier to history-taking and decision-making. These findings suggest that incapacity should not be presumed as a meaningful subset of residents can participate in shared decision-making. This argues for individualized assessment of capacity rather than the common assumption that NH residents, as a group, cannot meaningfully participate [9, 16, 22]. It is also consistent with evidence that structured penicillin allergy evaluation and delabeling can be performed safely in older inpatients, with high rates of success and minimal adverse events [23].

A second finding was a juxtaposition between resident and HCP perspectives toward delabeling. Residents commonly voiced uncertainty about their PALs and were conditionally open to evaluation when provided with clear explanations, reassurance, and a recommendation from a trusted clinician. Healthcare professionals, by contrast, frequently doubted the accuracy of PALs yet defaulted to avoidance, prescribing alternatives rather than verifying, citing safety, liability, and resource concerns. Although both groups conceptually supported delabeling, neither initiated it, and PALs tended to persist by inertia. This pattern is consistent with work in VA NH settings, where providers recognize the importance of allergy verification but worry about safety and resources [16]. Our findings extend the literature by showing that the same tension spans a broader NH population and can produce inaction even amid mutual conceptual support.

The interviews also distinguished knowledge gaps and implementation barriers. Limited familiarity with penicillin allergy evaluation and delabeling strategies was evident on both sides as residents reported little prior awareness of these options, and HCPs acknowledged limited training while expressing openness to education and willingness to engage if appropriately supported, suggesting knowledge deficits alone are not the principal barrier. Rather, structural constraints, such as staffing shortages, workflow limitations, reliance on third-party pharmacies, and the absence of clear ownership, with providers themselves disagreeing about who should be responsible for delabeling, emerged as more decisive. This contrasts with the VA study where leadership was prepared to enact policy and pharmacy leadership was positioned to champion penicillin allergy verification [16]. The clearer ownership and on-site pharmacy of an integrated system may explain why our non-VA NH experienced these structural barriers more acutely. These findings align with implementation science frameworks emphasizing integration into existing workflows, multidisciplinary collaboration, and standardized protocols [3, 5, 9, 10, 24]. In fragmented, resource-limited NH settings, interventions that depend on individual provider initiative are unlikely to be sustained.

Perceptions of specific strategies further informed feasibility. History-based assessment was viewed as the most practical approach and one that could be embedded in existing admission workflows. Views on testing modalities, however, were divided and reflected 2 distinct logics rather than a single preference. The same facility-level realities, including staffing, monitoring capacity, and access to emergency support, led providers to opposite conclusions about which modality was safer or more practical. This divergence suggests that no single testing modality will suit all facilities. It also implies that concerns about direct oral challenge may be disproportionate to its actual risk, consistent with evidence that low-risk or vague reactions can be safely addressed on the basis of history alone [4, 9] and that direct oral challenge carries low adverse event rates under appropriate protocols in older adults [7, 25]. Implementation in NH should therefore prioritize low-resource interventions, standardized history-taking, and risk stratification, while allowing facility-level flexibility in whether and how testing is added.

Finally, our findings underscore the importance of trust, communication, and stakeholder engagement. Although many HCPs expected residents and families to welcome delabeling, others cautioned that receptiveness cannot be assumed. Resistance may stem from prior adverse experiences, strong attachment to an existing label, or a sense that evaluation is not worth pursuing late in life. The single caregiver's refusal illustrated this, declining evaluation of their spouse's label outright. This accords with the VA study, whose larger sample of families and proxies likewise reported objection among proxies of cognitively impaired residents [16]. Because families and healthcare proxies often hold decision-making authority in NH, particularly when cognitive impairment is present, engaging them is essential, and delabeling efforts should be designed around shared decision-making rather than clinician initiative alone.

This study has several limitations. First, it was conducted in 5 NH within a single metropolitan region. Consistent with qualitative implementation research, our aim was analytic insight and transferability rather than statistical generalizability, and the determinants identified are meant to inform strategy design and hypotheses for subsequent testing rather than to estimate prevalence or effect size. Our design also cannot formally separate facility-level from provider-level sources of the divergence in testing opinions. Second, only 5 of 103 invited facilities permitted recruitment, reflecting well-documented challenges of conducting research at NH. Third, both facilities and individuals participated voluntarily, likely selecting for those already favorable toward antimicrobial stewardship. This would tend to understate barriers, as structural constraints predominated even among comparatively engaged participants. Fourth, a single interviewer conducted all interviews and led coding; the analysis is subject to potential interpreter bias. Despite second analyst review and positionality safeguards described in Methods, we did not perform independent double-coding, member checking, field notes, or maintain a formal audit trail, and residual bias cannot be excluded. Fifth, our rapid, deductive CFIR analysis prioritizes actionable determinants but may under-capture latent themes, and standardized information provided during interviews may have primed some responses, particularly regarding willingness to pursue evaluation may have been influenced by information. Sixth, only one physician participated. Unlike hospitals, NH typically rely on a small number of physicians, often contracted through third-party groups, who each cover multiple facilities, which limit the pool of eligible physicians. Physician perspectives may therefore be incompletely captured. To avoid overweighting this participant, we selected illustrative quotations across HCP roles wherever others expressed the same concept. Finally, caregiver perspectives were derived from a single participant and should be read as illustrative. Proxies were sought only for residents unable to participate directly, a small and difficult-to-recruit group that future studies should target with dedicated recruitment strategies.

## CONCLUSIONS

In summary, we identified key opportunities and challenges for implementing penicillin allergy evaluation and delabeling in NH settings. Many residents could engage meaningfully in discussions about their allergy and were conditionally willing to be evaluated, suggesting that the capacity to participate should be assessed individually rather than presumed absent, while recognizing that families and proxies will be important when cognitive impairment limits participation. Although residents and HCPs both recognized the potential value of delabeling, action was constrained primarily by structural barriers, workflow challenges, unclear ownership, and safety concerns rather than by a lack of interest. Successful delabeling strategies in NH are therefore likely to prioritize low-resource, history-based approaches integrated into existing workflows; define clear ownership; and emphasize education, trust, and shared decision-making with residents and families. Addressing these factors may enable broader and safer adoption of penicillin allergy delabeling and improve antibiotic use and outcomes in this high-risk population.

## Supplementary Material

ofag693_Supplementary_Data

## Supplementary Data

Supplementary materials are available at *Open Forum Infectious Diseases* online. Consisting of data provided by the authors to benefit the reader, the posted materials are not copyedited and are the sole responsibility of the authors, so questions or comments should be addressed to the corresponding author.
