# Supplementary material for: Penicillin Allergy Evaluation and Delabeling in Nursing Homes: An Implementation Science-Guided, Multi-stakeholder Qualitative Study
Source: Open Forum Infect Dis. 2026 Oct 23;13(11):ofag693. doi: 10.1093/ofid/ofag693 (PMC13736115; doi:10.1093/ofid/ofag693)
Supplement: Supplementary file 1 [file ofag693_supplementary_data.docx]

**Supplementary Appendix S1.** Consolidated criteria for reporting qualitative research (COREQ) checklist

| **Topic** | **Item No.** | **Guide Questions/ Description** | **Author Responses** |
| --- | --- | --- | --- |
| **Domain 1: Research team and reflexivity** | | | |
| *Personal Characteristics* | | | |
| Interviewer/facilitator | 1 | Which author/s conducted the interview or focus group? | KSF conducted interviews. |
| Credentials | 2 | What were the researcher’s credentials? E.g. PhD, MD | KSF is an infectious disease physician. |
| Occupation | 3 | What was their occupation at the time of the study? | The study team consists of a senior medical sociologist (AL) and three physicians trained in qualitative methods (KSF, MA, AW). |
| Gender | 4 | Was the researcher male or female? | KSF is identified as a man. |
| Experience and training | 5 | What experience or training did the researcher have? | KSF received formal implementation science education, including qualitative research from University of California, San Francisco. KSF also received training from AL on qualitative coding and analysis. AL holds a PhD in sociology and has qualitative methods expertise. AW and MA are physicians with qualitative research expertise. |
| *Relationship with participants* | | | |
| Relationship established | 6 | Was a relationship established prior to study commencement? | The study team had no relationship with interviewees prior to study commencement. The study team was not involved in the clinical care of the nursing home residents and caregivers. |
| Participant knowledge of the interviewer | 7 | What did the participants know about the researcher? e.g. personal goals, reasons for doing the research | KSF informed participants that he was part of a research team studying experiences and perspectives related to penicillin allergy evaluation and delabeling in nursing home setting. |
| Interviewer characteristics | 8 | What characteristics were reported about the inter-viewer/facilitator? e.g. Bias, assumptions, reasons and interests in the research topic | KSF has a professional interest in antimicrobial stewardship and penicillin allergy delabeling, and the team remained attentive to how this orientation might shape interpretation. |
| **Domain 2: Study design** | | | |
| *Theoretical framework* | | | |
| Methodological orientation and Theory | 9 | What methodological orientation was stated to underpin the study? e.g. grounded theory, discourse analysis, ethnography, phenomenology, content analysis | We used an applied qualitative approach with rapid thematic analysis. The interview guides and analysis were guided by the updated Consolidated Framework for Implementation Research (CFIR) with a focus on domains related to individuals, inner setting, outer setting, innovation, and implementation process, in order to capture key determinants of penicillin allergy evaluation and delabeling, including stakeholder knowledge and beliefs, organizational resources and workflows, external influences, and implementation processes within nursing home setting. |
| Sampling | 10 | How were participants selected? e.g. purposive, convenience, consecutive, snowball | The study information was disseminated by Massachusetts Department of Public Health through their nursing home listservs. KSF subsequently conducted direct outreach to interested facilities. Facility leadership provided permission for recruitment within participating nursing homes. KSF then recruited participants and screened interested individuals for eligibility before scheduling interviews.  We used purposive sampling to recruit three stakeholder groups:  1. Nursing home residents with a documented penicillin allergy label.  2. Caregivers of such residents.  3. Nursing home healthcare professionals, including physicians, advanced practice providers, nurses, nursing directors, pharmacists, and administrators (collectively HCPs). |
| Method of approach | 11 | How were participants approached? e.g. face-to-face, telephone, mail, email | Participants were approached either by phone or face-to-face for the initial study recruitment. They were then scheduled for an interview in person at a private space provided by the participating facility.  Participants received a $50 gift card for compensation. |
| Sample size | 12 | How many participants were in the study? | Total of 25 participants:   - 13 nursing home residents. - 1 caregiver. - 11 nursing home healthcare professionals. |
| Non-participation | 13 | How many people refused to participate or dropped out? Reasons? | 11 individuals screened declined to participate:   - 8 nursing home residents were unable to engage in meaningful communication. - 3 healthcare professionals were not interested. |
| *Setting* | | | |
| Setting of data collection | 14 | Where was the data collected? e.g. home, clinic, workplace | Data collection occurred at the participating facilities. |
| Presence of non-participants | 15 | Was anyone else present besides the participants and researchers? | None. |
| Description of sample | 16 | What are the important characteristics of the sample? e.g. demographic data, date | Interviews were conducted from July through December 2025. Demographic characteristics of participants are summarized in Table 1. Most nursing home residents were aged >60 years and were primarily White (79%). |
| *Data collection* | | | |
| Interview guide | 17 | Were questions, prompts, guides provided by the authors? Was it pilot tested? | Interviews were guided by interview guides with questions and prompts for each stakeholder group. Each interview guide was pilot tested.  Each interview guide began by exploring participants' understanding of and experiences with penicillin allergy label, including perceptions of allergy validity and impact on care. We then provided standardized information about penicillin allergy label and penicillin allergy evaluation/delabeling and asked participants about perceived benefits, risks, reactions, and willingness to pursue testing. We subsequently explored barriers, facilitators, and implementation strategies, with additional questions for healthcare professionals regarding organizational context and implementation readiness. We concluded each interview by asking participants whether there were additional perspectives or experiences they wished to share. |
| Repeat interviews | 18 | Were repeat interviews carried out? If yes, how many? | No. |
| Audio/visual recording | 19 | Did the research use audio or visual recording to collect the data? | Interviews were audio-recorded and professionally transcribed. All participants consented for audio recording. |
| Field notes | 20 | Were field notes made during and/or after the interview or focus group? | No. |
| Duration | 21 | What was the duration of the interviews or focus group? | 30 to 45 minutes. |
| Data saturation | 22 | Was data saturation discussed? | Formal data saturation was not a primary sampling criterion. |
| Transcripts returned | 23 | Were transcripts returned to participants for comment and/or corrections? | No. |
| **Domain 3: analysis and findings** | | | |
| *Data analysis* | | | |
| Number of data coders | 24 | How many data coders coded the data? | Three coders: KSF, MA, AL  KSF summarized participant responses and extracted representative quotations after each interview using an analytic matrix template. MA then reviewed the coding against the transcript to ensure accuracy. KSF, MA, and AL then iteratively refined themes through discussion and resolved discrepancies by consensus. |
| Description of the coding tree | 25 | Did authors provide a description of the coding tree? | We used a stakeholder-specific analytic matrix template to code data systematically and to support data reduction and analysis.  We use a rapid thematic analysis approach to identify responses most relevant to implementation strategy development. |
| Derivation of themes | 26 | Were themes identified in advance or derived from the data? | Themes were identified from the data. |
| Software | 27 | What software, if applicable, was used to manage the data? | Microsoft Excel. |
| Participant checking | 28 | Did participants provide feedback on the findings? | No. |
| *Reporting* | | | |
| Quotations presented | 29 | Were participant quotations presented to illustrate the themes/findings?  Was each quotation identified? e.g. participant number | Quotations are presented using deidentified participant ID for each stakeholder group. |
| Data and findings consistent | 30 | Was there consistency between the data presented and the findings? | The data presented were aligned and consistent with the findings, and supporting quotations are provided. |
| Clarity of major themes | 31 | Were major themes clearly presented in the findings? | Results were organized into five cross-cutting themes and divergent perspectives (minor themes) across stakeholders.  Each theme with its corresponding CFIR domains and constructs and representative quotations is presented in Table 2. |
| Clarity of minor themes | 32 | Is there a description of diverse cases or discussion of minor themes? | Diverse cases and minor themes are reported in the Divergent Perspectives section, including a minority of residents who were confident their labels were accurate, a caregiver who opposed evaluation (reflecting family- and proxy-related resistance), and a minority of healthcare professionals who trusted existing labels. |

Adopted from: Tong A, Sainsbury P, Craig J. Consolidated criteria for reporting qualitative research (COREQ): a 32-item checklist for interviews and focus groups. *Int J Qual Health Care*. 2007;19:349-57.

**Supplementary Appendix S2**. Semi-structured interview guides

a. **Interview guide for nursing home residents**

**Interview Guide for People with Reported Penicillin Allergy**

Participant ID _____________

Interview Date (MM/DD/YYYY) __________

**Introduction Script**

My name is (INSERT INTERVIEWER NAME). I am a researcher and physician at Tufts Medical Center. Thank you for deciding to be part of this interview. I am asking you to participate in this interview because you have an allergy to penicillin, an antibiotic used to treat infections. I would like to ask you questions about your penicillin allergy. There are no “right” or “wrong” answers to these questions.

To make sure that I have accurate responses from your interview, I would like to audio record our conversation. If you say no, I will take notes instead. I may ask you to pause or repeat something.

Do I have your permission to audio record this interview?	YES	NO

*Audio recorder turned on (circle):*	YES	NO

If you do not feel comfortable answering a question, it is okay for you to tell me that you do not want to answer. If there’s a question that you would prefer not to answer, you may simply say “pass.” You can also choose to stop this interview at any time and for any reason.

*Before we begin, do you have any questions?*

Time Interview Started: _________ AM / PM

Time Interview Ended: _________ AM / PM

**Section 1:**

To begin, I will ask you questions about your history of penicillin allergy:

| Interview Questions | Probes |
| --- | --- |
| Tell me how you first found out you had a penicillin allergy? | Do you remember what happened when you took penicillin?  What symptoms do you remember having?  Did you go to the doctor or hospital?  When did it happen? |
| How do you think having a penicillin allergy has affected your health? | What are your main concerns about having a penicillin allergy?  **OR**  It sounds like you think penicillin allergy has not impacted your health. Can you tell me more about that? |
| Tell me about the discussions you had with doctors and other people in medicine about your penicillin allergy | How did it make you feel to have these discussions?  **OR**  Tell me your thoughts on whether you should have had more discussions about your penicillin allergy. |

**Section 2:**

Research shows that about 1 in 10 people in the U.S. say they have a penicillin allergy. In Massachusetts nursing homes, that number is even higher, close to 1 in 4. In reality, only 1 in 100 are actually allergic.

Often, people think they're allergic because they had symptoms like diarrhea, headache, or tiredness, which aren't actually allergic reactions. Others may have had a rash as a child or don’t remember the details. Even when someone did have a true allergy in the past, about 80% outgrow it over time after 10 years.

| Interview Questions | Probes |
| --- | --- |
| What are your thoughts about this information? | Now that I have discussed this with you, do you remember any times that you may have heard this from your doctors or nurses before? |
| How does this information make you feel? |  |
| Has anyone ever told you that you might not have true allergy to penicillin? |  |

**Section 3:** Talking about Penicillin with Your Doctor

Next, I am going to talk about the process of a penicillin allergy evaluation. This process is called penicillin allergy delabeling. The first step is talking to patients about their penicillin allergy. Based on this process, it is possible that the doctor can confidently say you do not have a true allergy.

| Interview Questions | Probes |
| --- | --- |
| What do you think about this first step in the process? | If a doctor recommends that you to go through this process, how would that make you feel? |

**Section 4**: Testing for Penicillin Allergy

Some people might be eligible for something called an “oral challenge”. This is when a person who might have a penicillin allergy takes a dose of amoxicillin by mouth and is observed for about an hour. If there are no symptoms develop, the person is not allergic. This method is usually recommended for people with low-risk symptoms like itching, or a remote unknown history of penicillin allergy often start here.

| Interview Questions | Probes |
| --- | --- |
| What do you think about this next step? | If a doctor recommends that you to go through this process, how would that make you feel? |

**Section 5:** Skin Test

There is also something called a skin test. It’s for people with symptoms like rash, hives, or swelling. A small amount of penicillin product is put on the skin. If a raised, red area appears, it means you are allergic. If not, you move to the “amoxicillin challenge.” Penicillin allergy testing is supervised by a healthcare professional. The risk of a reaction is very small. If you do react, you will be given an oral antihistamine like Benadryl.

| Interview Questions | Probes |
| --- | --- |
| What do you think about this | If a doctor recommends that you to go through this process, how would that make you feel? |

**Section 6**: General Questions

Now that I have told you about this process, I will ask some general questions.

| Interview Questions | Probes |
| --- | --- |
| Have you heard about any of this information before? | Did you learn about testing from your doctor, TV, social media, or somewhere else? |
| How confident do you feel about the information you have just heard about penicillin allergy evaluation or testing? | What else would help you feel sure about the testing? |
| What do you think about having a penicillin allergy test to find out if you are truly allergic? | What would make you feel more comfortable about doing it?  What might stop you from doing it? |
| What would help make you feel more comfortable going through with a penicillin allergy evaluation or testing? | Would it help to have someone explain the process, or talk to you before and after the testing? |
| If a medical test showed you were not allergic to penicillin, how would that make you feel? |  |
| If a test showed you weren't allergic to penicillin, would you be willing to try penicillin again if the test said it was safe? | Tell me more about these thoughts. |

**Section 7:**

Thank you for answering these questions.

I have asked you a lot about penicillin allergy and testing. I want to ask you one more concluding question:

**Is there anything else you think is important to say about this topic that I haven’t asked you already?**

**Participant Self-Identification:**

(1.) Self-Identified Race __________________	(4.) Self-Identified Ethnicity ______________________
(2.) Self-Identified Gender ________________	(5.) Self-Reported Occupation ____________________
(3.) Self-Reported Level of Education ___________ (6.) Self-Reported Age _______
b. **Interview guide for caregivers**

**Interview Guide for Caregiver of Patients with Reported Penicillin Allergy**

Participant ID _____________

Interview Date (MM/DD/YYYY) __________

**Introduction Script**

My name is (INSERT INTERVIEWER NAME). I am a researcher and physician at Tufts Medical Center. Thank you for deciding to be part of this interview. I am asking you to participate in this interview because you are a caregiver for a patient with a penicillin allergy. I would like to ask you questions about the penicillin allergy of the person you care for. There are no “right” or “wrong” answers to these questions.

To make sure that I have accurate responses from your interview, I would like to audio record our conversation. If you say no, I will take notes instead. I may ask you to pause or repeat something.

Do I have your permission to audio record this interview?	YES	NO

*Audio recorder turned on (circle):*	YES	NO

If you do not feel comfortable answering a question, it is okay for you to tell me that you do not want to answer. If there’s a question that you would prefer not to answer, you may simply say “pass.” You can also choose to stop this interview at any time and for any reason.

*Before we begin, do you have any questions?*

Time Interview Started: _________ AM / PM

Time Interview Ended: _________ AM / PM

**Section 1:**

To begin, I will ask you questions about penicillin allergy of the person you care for:

| Interview Questions | Probes |
| --- | --- |
| Tell me how you first learned that the person you care for had a penicillin allergy. | Who told you about it? Was it a doctor, nurse, or someone else?  What were you told about the reaction? What symptoms were described?  Was any treatment given? Did they go to the doctor or hospital?  When did it happen? |
| How do you think penicillin allergy affects their health? | What are your main concerns about their penicillin allergy?  **OR**  It sounds like you think penicillin allergy has not impacted their health. Can you tell me more about that? |
| Tell me about any conversations you had with doctors or other people about the penicillin allergy of the person you care for. | How did it make you feel to have these discussions?  **OR**  Tell me your thoughts on whether you should have had more discussions about their penicillin allergy. |

**Section 2:**

Research shows that about 1 in 10 people in the U.S. say they have a penicillin allergy. In Massachusetts nursing homes, that number is even higher, close to 1 in 4. In reality, only 1 in 100 are actually allergic.

Often, people think they're allergic because they had symptoms like diarrhea, headache, or tiredness, which aren't actually allergic reactions. Others may have had a rash as a child or don’t remember the details. Even when someone did have a true allergy in the past, about 80% outgrow it over time after 10 years.

| Interview Questions | Probes |
| --- | --- |
| What are your thoughts about this information? | Do you remember any times that you may have heard this from the doctors or nurses before? |
| How does this information make you feel? |  |
| Has anyone ever told you that the person you care for might not have true allergy to penicillin? |  |

**Section 3:** Penicillin Allergy Evaluation and Delabeling in General

Next, I am going to talk about the process of a penicillin allergy evaluation. This process is called penicillin allergy delabeling. The first step is talking to patients about their penicillin allergy. Based on this process, it is possible that the doctor can confidently say you do not have a true allergy.

| Interview Questions | Probes |
| --- | --- |
| How comfortable would you feel if the provider asked you detailed questions about their penicillin allergy history? |  |

**Section 4**: Testing for Penicillin Allergy and Skin test

Some people might be eligible for something called an “oral challenge”. This is when a person who might have a penicillin allergy takes a dose of amoxicillin by mouth and is observed for about an hour. If there are no symptoms develop, the person is not allergic. This method is usually recommended for people with low-risk symptoms like itching, or a remote unknown history of penicillin allergy often start here.

There is also something called a skin test. It’s for people with symptoms like rash, hives, or swelling. A small amount of penicillin product is put on the skin. If a raised, red area appears, it means you are allergic. If not, you move to the “amoxicillin challenge.” Penicillin allergy testing is supervised by a healthcare professional. The risk of a reaction is very small. If you do react, you will be given an oral antihistamine like Benadryl.

| Interview Questions | Probes |
| --- | --- |
| How would you feel if the person you care for was recommended to have one of these testing? |  |

**Section 5**: General Questions

Now that I have told you about this process, I will ask some general questions.

| Interview Questions | Probes |
| --- | --- |
| Have you heard about any of this information before? | Did you learn about testing from your doctor, TV, social media, or somewhere else? |
| How confident do you feel about the information you have just heard about penicillin allergy evaluation or testing? | What else would help you feel sure about the testing? |
| What do you think about having a penicillin allergy test for the person you care for to find out if they are truly allergic? | What would make you feel more comfortable about doing it?  What might stop you from doing it? |
| What would help make you feel more comfortable with the person you care for going through a penicillin allergy evaluation or testing? | Would it help to have someone explain the process to you, or talk with you before and after the testing? |
| If a medical test showed that the person you care for was not allergic to penicillin, how would that make you feel? |  |
| If a test showed that the person you care for wasn’t allergic to penicillin, would you feel comfortable with them taking penicillin again if it was considered safe? | Tell me more about these thoughts. |

**Section 7:**

Thank you for answering these questions.

I have asked you a lot about penicillin allergy and testing. I want to ask you one more concluding question:

**Is there anything else you think is important to say about this topic that I haven’t asked you already?**

**Participant Self-Identification:**

(1.) Self-Identified Race __________________	(4.) Self-Identified Ethnicity ______________________
(2.) Self-Identified Gender ________________	(5.) Self-Reported Occupation ____________________
(3.) Self-Reported Level of Education ____________(6.) Self-Reported Age _______
c. **Interview guide for healthcare professionals**

**Interview Guide for Healthcare Professionals for Patients with Reported Penicillin Allergy**

Participant ID _____________

Interview Date (MM/DD/YYYY) __________

**Introduction Script**

My name is (INSERT INTERVIEWER NAME). I am a researcher and physician at Tufts Medical Center. Thank you for deciding to be part of this interview. I am asking you to participate in this interview because you are a healthcare professional caring for a patient with a penicillin allergy. I would like to ask you questions about your patient’s penicillin allergy. There are no “right” or “wrong” answers to these questions.

To make sure that I have accurate responses from your interview, I would like to audio record our conversation. If you say no, I will take notes instead. I may ask you to pause or repeat something.

Do I have your permission to audio record this interview?	YES	NO

*Audio recorder turned on (circle):*	YES	NO

If you do not feel comfortable answering a question, it is okay for you to tell me that you do not want to answer. If there’s a question that you would prefer not to answer, you may simply say “pass.” You can also choose to stop this interview at any time and for any reason.

*Before we begin, do you have any questions?*

Time Interview Started: _________ AM / PM

Time Interview Ended: _________ AM / PM

**Section 1:**

To begin, I would first like to understand how penicillin allergies are currently handled in your facility.

| Interviewer Questions | Probes |
| --- | --- |
| Tell me how allergies, including penicillin allergy, are usually reviewed or updated in your facility? | When do you review allergies at admission, during prescribing, or at other times?  Who is involved in that process? |
| Tell me what typically happens when a resident has a penicillin allergy label? | Tell me about any policies for penicillin allergy verification that exist? In particular, are there policies when the resident is cognitively impaired that encourage reaching out to family or caregivers? |
| Have you ever suspected that a penicillin allergy label on your patient might not be accurate? | How did you handle it? |
| On a scale of 1 to 10 (where 1 means “no impact at all” and 10 means “a major impact,”), how much do you think a penicillin allergy impacts a patient’s health? | Tell me about why you chose this number. |

**Section 2:** Taking about Penicillin Allergy in General

Research shows that about 1 in 10 people in the U.S. say they have a penicillin allergy. In Massachusetts nursing homes, that number is even higher, close to 1 in 4. In reality, only 1 in 100 are actually allergic.

Often, people think they're allergic because they had symptoms like diarrhea, headache, or tiredness, which aren't actually allergic reactions. Others may have had a rash as a child or don’t remember the details. Even when someone did have a true allergy in the past, about 80% outgrow it after 10 years.

| Interview Questions | Probes |
| --- | --- |
| What are your thoughts about this information? | How does this compare to what you previously knew about penicillin allergy? |

**Section 3:** Delabeling through Allergy History Taking

In many penicillin allergy evaluation protocols, the first step is taking a structured allergy history and discussing it with the resident and sometimes their caregivers. Providers may review the reported reaction, clarify details, and determine if the history is consistent with a true allergy. If it is not, the inaccurate label can be removed through a process known as penicillin allergy delabeling.

| Interview Questions | Probes |
| --- | --- |
| What do you think about this first step in the process? | How feasible would it be for staff in this facility to perform a structured allergy history or discussion with residents?  Who do you think would be best positioned to have these conversations (nurses, pharmacists, physicians)? |

**Section 4**: Oral Amoxicillin Challenge

Some residents with low-risk allergy histories, such as those with a mild skin rash, might be eligible for an oral challenge. The resident will take a small test dose of amoxicillin and is then watched closely for an hour. If no reactions happen, it shows they are not allergic and can be delabeled.

| Interview Questions | Probes |
| --- | --- |
| What do you think about this? | Do you think oral amoxicillin challenge can be implemented in your facility? Why or why not? |

**Section 5:** Penicillin Skin Test

Some residents with moderate to high-risk allergy histories, such as those with hives and anaphylaxis, a skin test may be done first before an oral challenge. This involves placing a small amount of penicillin under the skin to see if a reaction occurs. The test is done by trained staff with the right equipment, and the resident is monitored carefully throughout.

| Interview Questions | Probes |
| --- | --- |
| What do you think about this | Do you think skin testing can be implemented in your facility? Why or why not? |

**Section 6**: Targeted Questions on Strategies and Implementation

Now that I have told you about this process, I will ask some general questions.

| Interviewer Questions | Probes |
| --- | --- |
| What are some systems that you think could improve penicillin allergy evaluation? |  |
| How do you think residents or their families would respond to conversations about assessing penicillin allergy? |  |
| We have some ideas for specific strategies to support penicillin allergy delabelling. We would love to get your feedback |  |
| 1) First, let’s talk about the nurse champion. How do you see a nurse champion role to lead penicillin allergy evaluation and delabeling? | If not a nurse champion, who else in the facility might be well-positioned to lead this initiative? |
| 2) Next, we can talk about evaluation during admission intake. What do you think about including a few structured questions about penicillin allergy into the admission assessment? |  |
| 3) We can now talk about electronic health record flags. What do you think about using simple flags in the electronic health record to remind staff to review or update penicillin allergy information? | At what points in care (for example, admission, medication reconciliation, prescribing) would these prompts be most useful? |
| 4) Now, we can talk about the role of pharmacists. What do you think about pharmacists reviewing penicillin allergy labels as part of their medication review process and helping identify residents who could be considered for delabeling? |  |
| 5) Next, let’s talk about referral. What do you think about creating a standardized referral pathway to an allergy clinic for residents with a penicillin allergy label who may be candidates for delabeling? | What steps are usually involved in making a referral?  What do you think about telemedicine?  What do you think about once a week or once a month onsite evaluation by an allergist? |
| 6) What do you think about using short in-service education or simple handouts (like pocket cards or one-pagers) to give staff quick reminders about penicillin allergy delabeling? |  |
| 7) What do you think about an annual one-day evaluation and delabeling of all residents with a penicillin allergy? |  |
| 8) Are there any strategies we might have missed? |  |
| How does your facility put into place quality improvement initiatives | Who is involved in planning and implementation?  How do you think a penicillin allergy delabeling initiative would be received?  What would you need to feel prepared? (e.g., training, written protocols, external support) |

**Section 7:**

Thank you for answering these questions.

I have asked you a lot about penicillin allergy and testing. I want to ask you one more concluding question:

**Is there anything else you think is important to say about this topic that I haven’t asked you already?**

**Participant Self-Identification:**

(4.) Self-Identified Race __________________	(4.) Self-Identified Ethnicity ______________________
(5.) Self-Identified Gender ________________	(5.) Self-Reported Occupation ____________________
(6.) Self-Reported Level of Education ____________(6.) Self-Reported Age _______

**Supplementary Appendix S3:** Analytic matrix template for rapid thematic analysis

**a. Analytic matrix template for nursing home residents**

| **Interview section** | **CFIR domain: construct** | **Code** | **Subcode** | **Quotations** | **Summary** |
| --- | --- | --- | --- | --- | --- |
| **Section 1** | Individuals: Innovation Recipients (Capability). | Own Experience | Knowledge of own penicillin allergy |  |  |
|  | Individuals: Innovation Recipients (Need). |  | Perceived health impacts |  |  |
|  | Inner Setting: Access to Knowledge & Information. |  | Discussion with others |  |  |
| **Section 2** | Inner Setting: Access to Knowledge & Information.  Innovation: Evidence Base. | Accuracy of penicillin allergy | Reaction to general penicillin allergy information |  |  |
|  |  |  | Source of information |  |  |
|  | Individuals: Innovation Recipients (Motivation).  Innovation: Relative Advantage. |  | Reaction to general penicillin allergy information |  |  |
|  |  |  | Reaction to presumed inaccurate penicillin allergy |  |  |
| **Section 3, 4, 5** | Innovation: Complexity; Adaptability. | Penicillin allergy testing awareness and view in testing | History risk stratification |  |  |
|  |  |  | Oral challenge |  |  |
|  |  |  | Penicillin skin testing |  |  |
| **Section 6** | Inner Setting: Access to Knowledge & Information. | Evaluation | Exposure to testing info |  |  |
|  | Inner Setting: Available Resources; Access to Knowledge & Information.  Implementation Process: Engaging. |  | Support needs to feel confident in testing |  |  |
|  | Individuals: Innovation Recipients (Motivation). |  | Willingness to act |  |  |
|  | Inner Setting: Available Resources. |  | Support needs to feel prepared to go through testing |  |  |
|  | Individuals: Innovation Recipients (Motivation; Need). | Delabeling results | Reaction on delabeling result |  |  |
|  |  |  | Act on delabeling result |  |  |
| **Section 7** | - | **-** | Other thoughts |  |  |

**b. Analytic matric template for caregivers**

| **Interview section** | **CFIR domain: construct** | **Code** | **Subcode** | **Quotations** | **Summary** |
| --- | --- | --- | --- | --- | --- |
| **Section 1** | Individuals: Innovation Recipients (Capability). | Own Experience | Ability to relay accurate history |  |  |
|  | Individuals: Innovation Recipients (Need). |  | Perceived health impacts |  |  |
|  | Inner Setting: Access to Knowledge & Information. |  | Discussion with others |  |  |
| **Section 2** | Inner Setting: Access to Knowledge & Information.  Innovation: Evidence Base. | Accuracy of penicillin allergy | Exposure to general penicillin allergy information |  |  |
|  |  |  | Source of information |  |  |
|  | Individuals: Innovation Recipients (Motivation).  Innovation: Relative Advantage. |  | Reaction to information |  |  |
|  |  |  | Reaction to inaccurate penicillin allergy |  |  |
| **Section 3, 4, 5** | Innovation: Complexity; Adaptability. | Penicillin allergy testing awareness and view in testing | History risk stratification |  |  |
|  |  |  | Oral challenge |  |  |
|  |  |  | Penicillin skin testing |  |  |
| **Section 6** | Inner Setting: Available Resources; Access to Knowledge & Information. | Evaluation | Exposure to testing info |  |  |
|  | Inner Setting: Available Resources; Access to Knowledge & Information.  Implementation Process: Engaging. |  | Support needs to feel confident in testing |  |  |
|  | Individuals: Innovation Recipients (Motivation). |  | Willingness to act |  |  |
|  | Inner Setting: Available Resources. |  | Support needs to feel prepared to go through testing |  |  |
|  | Individuals: Innovation Recipients (Motivation; Need). | Delabeling result | Reaction on delabeling result |  |  |
|  |  |  | Act on delabeling result |  |  |
| **Section 7** | - | **-** | Other thoughts |  |  |

**c. Analytic matrix template for healthcare professionals**

| **Interview section** | **CFIR domain: construct** | **Code** | **Subcode** | **Quotations** | **Summary** |
| --- | --- | --- | --- | --- | --- |
| **Section 1** | Inner Setting: Structural Characteristics.  Individuals: Innovation Deliverers (Motivation).  Outer Setting: Partnerships and Connections. | Current practice | Process to review or update penicillin allergy |  |  |
|  |  |  | Actions taken for penicillin allergy |  |  |
|  |  |  | Skepticism or Trust in penicillin allergy |  |  |
|  | Individuals: Innovation Recipients (Need). | Impacts of penicillin allergy | - |  |  |
| **Section 2** | Individuals: Innovation Deliverers (Capability; Motivation).  Innovation: Evidence Base. | Accuracy of penicillin allergy | Exposure to this information |  |  |
| **Section 3** | Inner Setting: Structural Characteristics; Available Resources.  Innovation: Relative Advantage; Complexity, Adaptability. | History risk stratification | - |  |  |
| **Section 4** |  | Oral challenge | - |  |  |
| **Section 5** |  | Penicillin skin testing | - |  |  |
| **Section 6** | Inner Setting: Compatibility  Implementation Process: Planning. | Proposed strategies | - |  |  |
|  | Individuals: Innovation Recipients (Motivation).  Implementation Process: Engaging. | Resident & Family Receptiveness | - |  |  |
|  | Implementation Process: Planning; Engaging.  Individuals: Implementation Leads.  Outer Setting: Partnerships and Connections. | Proposed strategies for delabeling | Nurse champions |  |  |
|  |  |  | Verification using structured questions during admission intakes |  |  |
|  |  |  | EHR flagging |  |  |
|  |  |  | Verification by pharmacists during their medication review |  |  |
|  |  |  | Referral to specialist |  |  |
|  |  |  | Onsite allergist evaluation on a regular basis |  |  |
|  |  |  | Telemedicine referral |  |  |
|  |  |  | Short in-service education, simple handouts |  |  |
|  |  |  | Annual one-day penicillin allergy evaluation and delabeling |  |  |
|  |  |  | Others |  |  |
|  | Implementation Process: Teaming.  Individuals: High-level Leaders; Implementation Leads. | QI implementation | People involved in planning and implementation |  |  |
|  | Inner Setting: Relative Priority. |  | Receptiveness of delabeling QI initiatives |  |  |
|  | Inner Setting: Available Resources. Access to Knowledge & Information. |  | Support needs to feel prepared |  |  |
| **Section 7** | - | **-** | Other thoughts |  |  |
